# Barriers to deprescribing anti-depressants in a primary care setting; an observational study - Are SSRI drugs of dependence and should these be re classified as schedule 3 drugs

**DOI:** 10.1192/j.eurpsy.2023.1748

**Published:** 2023-07-19

**Authors:** A. Patel, R. Rasheed

**Affiliations:** Medicine, Rigg Milner Medical, East Tilbury, United Kingdom

## Abstract

**Introduction:**

There has been an exponential rise in SSRI prescribing, between 2021 and 2022 there was a 5% increase. The majority of SSRI prescription initiation takes place in primary care. This is a national trend and mirrored internationally.

**Objectives:**

The study was undertaken to understand barriers to deprescribing both prescribing clinicians and patients and the potential of dependence caused by continued prescribing of SSRI and SNRI. We wanted to understand deprescribing challenges and both clinical anxiety and difficulty experienced and see if there is a correlation with the Drug use screening tool (DUST) tool.

**Methods:**

Those patients who were stable on SSRI were offered lowering of dose and deprescribing as part of routine medication reviews. During medication reviews patients were asked about willingness to embark on a deprescribing schedule. Patients were screened during medication reviews on the DUST to see if this can be used to predict difficulty with deprescribing. We designed a deprescribing difficult questionnaire to assess the difficulty experienced by clinicians during a deprescribing consultation. We were able to study the is a correlation with DUST scores and Clinician experience of difficulty and challenge in deprescribing of the SSRI and SNRI.

**Results:**

Current alcohol drinkers and smokers were less likely to deprescribe from their antidepressants. Clinicals should do a risk assessment using the DUST screening tool checking for risk of dependence. There is an R value of 0.1586 (P-value is 0.018848) between the correlation of patients increased length time and increased DUST score causing an increased risk of dependence. The average R-score across the three practices between patient’s length of time being on medications and their DUST score is R= 0.18705 (P value of 0.01)

**Conclusions:**

Inability to access IAPT therapies, shortened length of CBT sessions and lack of post IAPT support caused poor patient experience and contributed to reluctance to re-engage with IAPT services. Both cascade and incremental prescribing following lower doses without documentation of the limitations of medication results in unrealistic expectations generated from the prescribing. There is positive correlation between length of SSRI and SNRI prescribed, DUST scores, and Clinical challenge scores with patients’ unwillingness to be deprescribed. Patients should have a DUST score review prior to having been put onto any antidepressant as there is a potential link between increased DUST score and lower chances of willingness to deprescribe. Clinicians need to counsel patient of risk of dependence. The likelihood is these are drugs of dependence and clinicians should counsel patients of these risks and given review dates and offered deprescribing.

**Disclosure of Interest:**

None Declared

